# Identification and Functional Analysis of SabHLHs in *Santalum album* L.

**DOI:** 10.3390/life12071017

**Published:** 2022-07-08

**Authors:** Ting Zhang, Xiaohong Chen, Yuping Xiong, Meiyun Niu, Yueya Zhang, Haifeng Yan, Yuan Li, Xinhua Zhang, Guohua Ma

**Affiliations:** 1 Guangdong Provincial Key Laboratory of Applied Botany, South China Botanical Garden, The Chinese Academy of Sciences, Guangzhou 510650, China; zhangting13jmk@163.com (T.Z.); chenxiaohong@scbg.ac.cn (X.C.); xiongyuping@scbg.ac.cn (Y.X.); niumeiyun@scbg.ac.cn (M.N.); tulipazhyy@163.com (Y.Z.); liy@scbg.ac.cn (Y.L.); xhzhang@scbg.ac.cn (X.Z.); 2 University of the Chinese Academy of Sciences, Beijing 100049, China; 3 Cash Crop Institute of Guangxi Academy of Agricultural Sciences, Nanning 530007, China; gstsyhf@163.com

**Keywords:** bHLH transcription factor, dual luciferase, gene cloning, sandalwood, *SaSSy*, *SaCYP736A167*, subcellular localization, yeast one-hybridization, dual luciferase activity

## Abstract

*Santalum album* L., a semi-parasitic evergreen tree, contains economically important essential oil, rich in sesquiterpenoids, such as (*Z*) α- and (*Z*) β-santalol. However, their transcriptional regulations are not clear. Several studies of other plants have shown that basic-helix-loop-helix (bHLH) transcription factors (TFs) were involved in participating in the biosynthesis of sesquiterpene synthase genes. Herein, bHLH TF genes with similar expression patterns and high expression levels were screened by co-expression analysis, and their full-length ORFs were obtained. These bHLH TFs were named *SaMYC1*, *SaMYC3*, *SaMYC4*, *SaMYC5*, *SabHLH1*, *SabHLH2*, *SabHLH3*, and *SabHLH4*. All eight TFs had highly conserved bHLH domains and *SaMYC1*, *SaMYC3*, *SaMYC4*, and *SaMYC5*, also had highly conserved MYC domains. It was indicated that the eight genes belonged to six subfamilies of the bHLH TF family. Among them, SaMYC1 was found in both the nucleus and the cytoplasm, while SaMYC4 was only localized in the cytoplasm and the remaining six TFs were localized in nucleus. In a yeast one-hybrid experiment, we constructed decoy vectors pAbAi-SSy1G-box, pAbAi-CYP2G-box, pAbAi-CYP3G-box, and pAbAi-CYP4G-box, which had been transformed into yeast. We also constructed pGADT7-*SaMYC1* and pGADT7-*SabHLH1* capture vectors and transformed them into bait strains. Our results showed that *SaMYC1* could bind to the G-box of *SaSSy,* and the *SaCYP736A167* promoter, which *SaSSy* proved has acted as a key enzyme in the synthesis of santalol sesquiterpenes and *SaCYP450* catalyzed the ligation of santalol sesquiterpenes into terpene. We have also constructed pGreenII 62-SK-*SaMYC*1, pGreenII 0800-LUC-*SaSSy* and pGreenII 0800-*LUC*-*SaCYP736A167* via dual-luciferase fusion expression vectors and transformed them into *Nicotiana benthamiana* using an *Agrobacterium*-mediated method. The results showed that *SaMYC*1 was successfully combined with *SaSSy* or *SaCYP736A167* promoter and the *LUC/REN* value was 1.85- or 1.55-fold higher, respectively, than that of the control group. Therefore, we inferred that *SaMYC1* could activate both *SaSSy* and *SaCYP736A167* promoters.

## 1. Introduction

Transcription factors (TFs) are key regulatory elements in plants that often bind to *cis*-acting elements (CAEs) in the promoter region upstream of a gene, and regulate its expression. They typically had four functional regions, a transcriptional regulatory region, a nuclear localization signal region, an oligomerization site region, and a DNA-binding region [1]. The amino-acid sequences of TF DNA-binding region determine its family, such as basic-helix-loop-helix (bHLH), MYB, WRKY, bZIP, MADs, TCP, AP2/ERF, or other families of TFs. Many TFs were involved in plant growth and development, secondary metabolism, stress resistance and other processes, but the TFs involved in regulating synthesis of sesquiterpenes usually fell into four families: AP2/ERF, bHLH, MYB, and WRKY [2,3,4]. Among them, bHLH TFs were the second largest family of TFs in plants after the MYB TFs, and they had a highly conserved domain that was divided into two regions—an alkaline region located at the N-terminus, which consisted of 15–20 amino acids, and another α helix 1-ring-α helix 2 region, located at the C-terminus, which consisted mainly of hydrophobic amino acids [5]. This domain consisted of about 60 amino acids, 25 of which were conserved residues, five were in the alkaline region, six were in the first spiral region, two were in the ring, and another 12 were in the second spiral region [6]. Alkaline regions could bind to the E-box (5′-CANNTG-3′) and the G-box (5′-CACGTG-3′) in DNA sequences [7]. The α-helix 1-cyclic-α helix 2 region contained many hydrophobic amino acids, and in order to be functional, it often formed homo- or heterodimers [8].

bHLH TFs were widely involved in the growth and development of plants. S1PRE2, a bHLH TF was highly expressed in immature green *Solanum lycopersicum* fruits after induction by gibberellic acid (GA_3_), and as the *S1PRE2* gene was silenced, fruits became smaller and pericarps became thinner, indicating that S1PRE2 was a positive regulator during fruit development [9]. bHLH TFs formed complexes with MYB TFs, activated the expression of key genes that regulated stamen development, seed germination, and seedling development in *Arabidopsis thaliana* [10]. bHLH TFs SPEECHLESS (SPCH), MUTE, FAMA, and ICE/SCREAM (SCRM) co-regulated the formation of plant stomata via signal transduction [11,12]. The bHLH-like TF LAX/ba1 co-regulated branching and inflorescence branching with GLAS family members Ls, LAS, and MOC1, and an R2R3-type MYB family member Bl [13].

Signal transduction is a very important process in plants because their response to external stimuli takes place via signal transduction. bHLH TFs played a key negative regulatory role in plant pigment signal transduction. They were also involved in plant hormone signal transduction [14]. Three bHLH TFs (*BEE1*, *BEE2* and *BEE3*) were regulators required for the early response of *A. thaliana* brassinosteroid (BR), as demonstrated by their mutants *bee1*, *bee2*, and *bee3* [15]. In *A. thaliana*, the bHLH TF *AtMYC2* upregulated an abscisic acid (ABA)-inducible gene, while a mutant of *AtMYC2* downregulated an ABA-inducible gene, demonstrating that it acted as a positive regulator in ABA-induced gene expression [16]. *AtMYC2* was also involved in the signal transduction pathway of Jasmonate-ZIM, which acted as a transcriptional inhibitor [17]. In *Malus pumila*, *MdbHLH3* activated the transcription of genes that regulated ethylene biosynthesis (*MdACO1*, *MdACS1*, and *MdACS5A*), thereby promoting the synthesis of ethylene [18]. The bHLH TF *PIF4* played a major role in multiple signal integration during plant growth regulation, serving as a positive regulator in cell elongation, and its activity was regulated by various environmental signals and hormonal signals including GA_3_, auxin, and BR, as well as light and temperature, both transcriptionally and post-translationally [19]. In *Oryza sativa*, nuclear localization of the TF *OsbHLH073* was involved in regulation of plant height, and internodal and panicle elongation by downregulating the biosynthesis of GA_3_ [20]. High temperatures might increase both epidermal *PIF4* transcription and the epidermal *PIF4* DNA-binding ability in *A. thaliana* [21].

*Santalum album* L. is a semi-parasitic tree that belongs to the Santalaceae family. It has a high economic value, which is mainly reflected in its heartwood, which is often used as a raw material for carving crafts, and it is often made into incense commonly used in perfume, while sandal essential oil extracted from its heartwood has displayed anti-cancer [22,23], antioxidant [24], anti-inflammatory and analgesic [25,26] properties, and has been used in the treatment of skin diseases [27,28]. The main components of sandal essential oil are α- and β-santalol [29]. Therefore, it is necessary to understand biosynthesis of the main sandal sesquiterpenes.

In recent years, an increasing amount of research has been dedicated to synthesis of sandal sesquiterpenes, which were mainly synthesized by the mevalonic acid pathway [30]. *SaSSy* and its homologous genes *SauSSy* and *SpiSSy* regulated synthesis of terpenoids such as α- and β-santalol, while the strongest regulatory function shown by *SaSSy*, and *SaSSy* acted as a key enzyme in synthesis of sandal sesquiterpenes [30]. SaCYP450 family enzymes catalyzed the ligation of sandal sesquiterpenes into terpene [31]. Among them, *SaCYP736A167* converted α- and β-santalol into (*Z*)-α- and (*Z*)-β-santalol [30,32]. The farnesyl diphosphate synthase gene *SaBS* was cloned from *S. album*, and it encoded an enzyme necessary for catalytic synthesis of the substrates, such as (*E*, *E*)-farniki pyrophosphate [33]. Three new terpene synthase (*TPS*) genes, *SaTPS1*, *SaTPS2* and *SaTPS3*, were isolated from *S. album*: while *SaTPS2* and *SaTPS3* catalyzed synthesis of (*E*)-α-bergamotene, (*E*)-β-farnesene and β-bisabolene, *SaTPS1*, *SaTPS2* and *SaTPS3* responded to hormones and abiotic stresses [34]. A *TPS* gene located in chloroplasts and the cytoplasm was isolated from *S. album*, the enzyme encoded by this gene mainly catalyzed synthesis of linalool and nerolol, which were secondary components of sandal essential oil, while this gene responded to abiotic stress [35]. In recent years, many studies have focused on upstream regulatory genes of key enzyme genes in the biosynthetic pathway of sandal oil, such as the *SaAACT* and *SaHMGS* genes, which regulated the synthesis of important substrates, and whose function was verified in yeast by complementation experiments [36,37]. *SaDXR* was a 1-deoxy-D-xylulose-5-phosphate reductoisomerase (DXR), cloned from *S. album*, which played an important role in the biosynthesis of photosynthetic pigments and shifted the flux to sesquiterpenoids [38].

The transcriptional regulation of sesquiterpenes by bHLH TFs in *S. album* has not been reported. The objective of this work was to identify bHLH TF genes in *S. album* transcriptome. The physicochemical properties were determined; bioinformatics and subcellular localization analyses were also performed. To explore whether these bHLH TF genes were involved in the expression of key enzyme genes that regulated the synthesis of santalol, some promoters of key enzyme genes (*SaSSy* and *SaCYP450*) were explored by yeast one-hybridization and dual-luciferase experiments. Our findings will provide a theoretical basis for additional studies of bHLH TFs, to assess their regulation of the synthesis of sandal sesquiterpenes.

## 2. Materials and Methods

Plant materials: The material used in this experiment included a 10-year-old *S. album* tree, which planted in the sandalwood research base of South China Botanical Garden of the Chinese Academy of Sciences, Guangzhou. Wild-type *Arabidopsis* seeds were preserved and grown in incubators at 22–23 °C in the Lab, 16-h photoperiod, 100 µmol m^−2^ s^−1^. *Nicotiana benthamiana* was grown at day/night 16-h photoperiod of 28/26 °C, 80 µmol m^−2^ s^−1^, in an incubator used for subsequent transient expression.

### 2.1. Reagents

Kits: 1% agarose gel DNA recovery kit, plasmid medium volume kit were purchased from Magen BioTech Co., Ltd. (Guangzhou, China). A dual luciferase activity assay kit was purchased from Promega (Beijing, China). Yeast one-hybrid kits and yeast ligation kits were purchased from Clontech (Terra Bella Avenue Mountain View, CA, USA).

Enzymes: 10× loading buffer, DL 2000 DNA Marker, pMD18-T, rTaq enzyme, T4 ligase, In-Fusion HD enzyme premix and various restriction nucleic acid endonucleases were purchased from TakaRa Bio Inc. (Dalian, China); KOD FX was purchased from OYO TBO (Osaka, Japan); and 2× Flash PCR MasterMix (Dye) were purchased from Kangwei Century Co (Beijing, China).

Culture medium: The components of the LB medium, yeast extract, tryptone, and sodium chloride were purchased from Oxoid Biological Company and Aladdin Biological Company, respectively.

Other reagents: Cellulase Cellulose R10 and pectinase Macerozyme used for subcellular localization were purchased from Yakult Honsha in Japan; Bovine Serum Protein (BSA) was purchased from Sigma (Merck KGaA, Darmstadt, Germany).

Vector and Escherichia coli lines: The subcellular localization vector pSAT6-EYFP-N1 was preserved by our laboratory; the *E. coli* DH5α line was purchased from Shanghai Vidi Biotechnology Co., Ltd. (Shanghai, China). Guangzhou Qingke Biotechnology Co., Ltd. (Guangzhou, China) provided the service for primer synthesis and sequencing.

### 2.2. Screening and Cloning of bHLH Transcription Factors

Based on our group’s existing sandal-tree-transcriptome data and the research achieved [3,35], eight bHLH TFs (SabHLHs) with similar expression patterns (more expression in heartwoods than expression in sapwoods) and similar expression patterns to *SaSSy* and *SaCYP736A167*, which regulated sandal oil biosynthesis, were screened by co-expression analysis. Mixed cDNA of the stems and leaves from the 10 years old sandal tree was used as template. PCR amplifications were carried out with TaKaRa rTaq enzyme and corresponding primers. PCR products were separated by electrophoresis agarose gel (1%) electrophoreses and recovered using the gel recovery kit (Meiji Biotech) according to the instructions. The purified PCR product was ligated overnight with T4 ligase with pMD18-T vector and transformed into *E. coli*. Single colonies containing the fragment of interest were picked and inoculate to liquid LB medium containing Amp antibiotics, incubated (37 °C, 200 rpm) for 12 h. The *E. coli* solution was sequenced in Qingke Biotech.Plasmids were extracted using plasmid small lifting kit (Meiji Biotech) according to the instructions. Eight TF ORF plasmids were then obtained (Supplementary Table S1). The ORF sequences of these eight TFs were submitted to NCBI for the registration numbers (Supplementary Table S2).

### 2.3. Bioinformatics Analysis of SabHLHs

Based on the sequence of SabHLHs, the amino acid length, molecular weight, isoelectric point, instability coefficient and mean hydrophilicity of these eight TFs were analyzed using the online website ExPASY (https://web.expasy.org/protparam/) (accessed on 21 February 2021). After translated by DNAstar editing, the conservative domain (Motif) of SabHLHs was analyzed using the online website MEME (http://meme-suite.org/tools/meme) (accessed on 21 February 2021) and graphed with TBtools software (https://www.tbtools.com/) (accessed on 21 February 2021) [16]. MEME parameters were set according to our previous work [3]. Protein sequences of bHLH from the pattern plant *A. thaliana* were downloaded from Phytozome (https://phytozome.jgi.doe.gov/pz/portal.html) database (accessed on 26 November 2020); multi-sequence alignment analysis was performed using ClustalX 2.0 (Supplementary Table S3). A systematic evolutionary tree was constructed using the Neighbor-Joining method in MEGA 7.0 [39], where the number of bootstraps was set to 1000.

### 2.4. Subcellular Localization Analysis 

pSAT6-EYFP-N1 plasmid was digested by *Bam*HI and *Eco*RI restriction endonucleases. TF fragments which removed stop codon were amplified using TaKaRa’s KOD FX and then cloned into pSAT6-EYFP-N1 vector by homologous recombinant ligase (TaKaRa). The recombinant vectors were transformed into *E. coli*. Positive colones were sequenced and plasmids were extracted.

We bathed 10 mL enzymatic solution for 10 min and cooled to room temperature, then added sterilized 100 μL CaCl_2_ (1.0 M) and 100 μL Bovine serum albumin (BSA). We then selected *A. thaliana* that was growing well and teared off the epidermis of *Arabidopsis* leaves with scotch tape and put them into a Petri dish containing the above enzymatic solution, and then incubated at 50 rpm at 22 °C for 3 h under weak light conditions. Slowly, we added W5 solution (150 mL NaCl, 125 mL CaCl_2_, 5 mM KCl, 2 mM ES, osmolarity 550–580 mOsm), pH5.7 (KCl), then stored at 4 °C; the amount of W5 solution was added depending on the number of cells; the final solution color with light green was preferred. We suspended the pellet gently; Microscopic examination to ensure the integrity and concentration of protoplasts was appropriate. No-load pSAT6-EYFP-N1 was used as a control.

### 2.5. Interaction Detection of Transcription Factors SaMYC1, SabHLH1 and G-Box Elements 

Eight transcription factors (*SaMYC1*, *SaMYC3*, *SaMYC4*, *SaMYC5*, *SabHLH1*, *SabHLH2*, *SabHLH3*, *SabHLH4*) were constructed into pGADT7-AD and transformed to the Y1H Gold strain to express the capture protein. We inoculated the well-grown single colonies into 3 mL YPDA liquid medium and shook to OD 0.2, then diluted with 0.9% NaCl solution for 100 fold to an OD value at 0.002, then took 4.5 μL of dots in SD/-Leu/AbA 0 ng/mL and SD/-Leu/AbA 200 ng/mL media, respectively.Sequences of *SaSSy* and *SaCYP736A167* promoters were submitted to the online software plantCARE (http://bioinformatics.psb.ugent.be/webtools/plantcare/html/) (SaSSy and SaCYP736A167 were both accessed on 11 December 2019) for G-Box and E-Box prediction.

*SaSSy* promoter contained G-Box upstream and downstream of the small fragment *SSy1G*. *SaCYP736A167* promoter contained G-Box 10bp upstream and downstream of small fragments *CYP1G*, *CYP2G*, *CYP3G*, *CYP4G*, respectively. The synthesis sequences by Qingke Biotech. Com. were shown in the Supplementary Table S4. *pAbAi-SSy1G*-Box, *pAbAi-CYP1G*-box, *pAbAi-CYP2G*-box, *pAbAi-CYP3G*-box and *pAbAi-CYP4G*-box plasmid were integrated into Y1H Gold yeast strain after restriction endonuclease BstbI monoenzyme cleavage. Positive single colony was shaken in 3 mL YPDA liquid medium to OD 0.2, and then adjusted the OD value to 0.002 with 0.9% NaCl solution, and 100 μL was screened for the lowest AbA inhibition concentrations in SD/-Ura media with different AbA concentrations.

AD-SaMYC1 and the negative control were transformed into wild type bait strains and mutant element bait strains. The transformed strain was first cultured on SD/-Leu medium that contained no AbA, after the colonies grew up, a well-grown monoclonal was picked. As the YPDA liquid medium was shaken to a bacterial liquid OD value of 0.2, and diluted with 0.9% NaCl solution to 100-fold to an OD value at 0.002, 4.5 μL of liquid was taken on the medium of SD/-Leu with corresponding concentration of AbA. After this was incubated at 30 °C for 3–5 d, the results were obtained.

### 2.6. SaMYC1 Activated SaSSy and SaCYP736A167 Promoter Activity

In order to further determine the regulatory effect of *SaMYC1* on *SaSSy* and *SaCYP736A167* genes, the transcription factor *SaMYC1* was constructed on a pGreenII 62-SK vector driven by a 35S promoter as an effective carrier. *SaSSy* and *SaCYP736A167* promoter sequences were constructed on a reporter vector containing *REN* gene and *LUC* gene pGreenII 0800-LUC vector as a reporter vector, which renilla luciferase gene *REN* was initiated by a 35S strong promoter on the vector, so renilla luciferase activity was used as a reference. The firefly luciferase gene was initiated and expressed by the *SaSSy* promoter. The transcription factor vector and promoter vector were transformed into *Agrobacterium* competent cell GV3101, respectively, which the OD value of bacterial fluid was 0.6–0.8, and then transformed into *N. benthamiana* leaves according to the ratio of transcription factor: promoter = 10:1 (*v*:*v*). After co-incubation for 2 days under normal conditions, the effect of *SaMYC1* on *SaSSy* promoter was determined by detecting the chemiluminescence values of firefly luciferase and renilla luciferase. The ratio of no-load pGreenII 62-SK+ *SaSSy*-0800-LUC, pGreenII 62-SK + *SaCYP736A167*-0800-LUC was used as the control, and the ratio of firefly luciferase chemiluminescence value divided renilla luciferase chemiluminescence value was *LUC/REN.*

### 2.7. Statistical Analysis

The experiment statistics were analyzed using IBM SPSS 19.0 (IBM Corp., Armonk, NY, USA), and Duncan’s multiple range test at *p* ≤ 0.05 to denote significant differences between the means. Different letters indicated a significant difference. All data represented three biological replicates of mean ± standard errors (SE).

## 3. Results

### 3.1. Cloning SabHLHs ORFs

**Figure 1:**
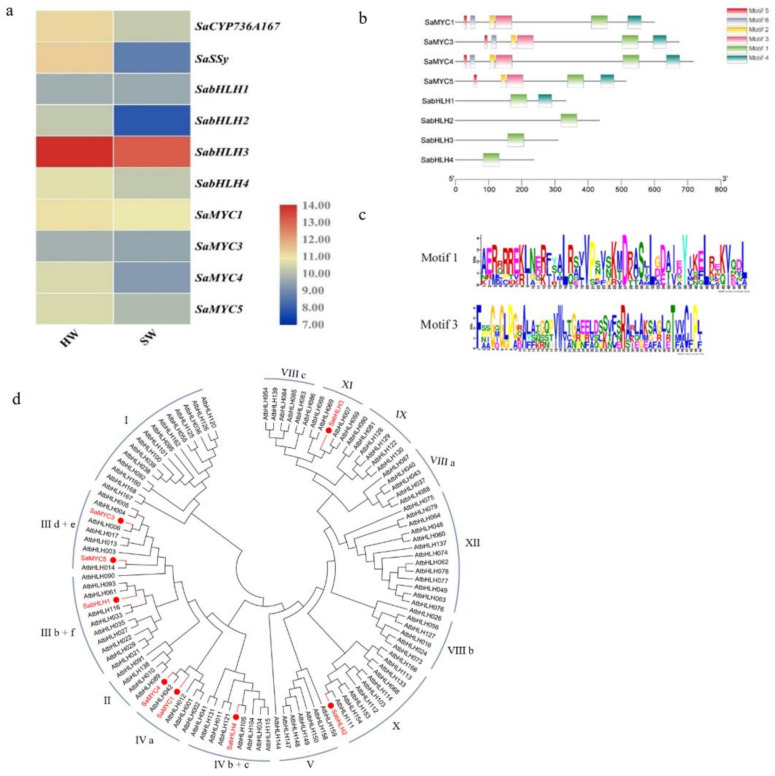
Sequence analysis of bHLH transcription factors and their co-expression patterns with *SaSSy* and *SaCYP736A167* in *Santalum album*. (**a**) Expression levels of bHLH transcription factors, *SaSSy* and *SaCYP736A167*. 10-year-old sandal tree was sampled. The heat map was generated based on log2-transformed count value from FPKM of transcriptome data using TBtools. HW: heartwood; SW: sapwood; (**b**) Schematic diagram of SabHLH motifs; (**c**) Motif 1 and Motif 3 domains; Motif 1 represents the bHLH domain, Motif 3 represents the MYC domain; (**d**) Phylogenetic analysis of SabHLH proteins in *Santalum album*. The bHLH transcription factors of *Santalum album* (marked with red) and *Arabidopsis thaliana* (black) were aligned by ClustalX 2.0, and the NJ (Neighbour-Joining) tree was constructed using MEGA 7.0 with 1000 bootstrap replicates.

Eight bHLH TFs were screened and named *SaMYC1*, *SaMYC3*, *SaMYC4*, *SaMYC5*, *SabHLH1*, *SabHLH2*, *SabHLH3*, and *SabHLH4* (Supplementary Tables S1–S3). A heat map was generated based on transcriptome data (Figure 1a). TF open reading frames (ORFs) were amplified by RT-PCR and then electrophoresed on a 0.1% agarose gel.

### 3.2. Analysis of the Physicochemical Properties of SabHLH Proteins

The amino acid length of these eight TFs was 235–716 aa and the molecular weight (MW) was 25.87–77.72 kDa. Most of these proteins were acidic; the isoelectric point (pI) of seven proteins was less than 7.0, and the average hydrophilic value of these proteins was less than 0, indicating that they were all hydrophobic. The instability coefficient of these proteins was greater than 40, suggesting that they were unstable proteins (Table 1).

### 3.3. Conservative Motif Analysis of SabHLH Proteins

All eight TFs had highly conserved bHLH domain Motifs (Figure 1b). SaMYC1, SaMYC3, SaMYC4, and SaMYC5 also had highly conserved MYC domains (Figure 1c).

### 3.4. Phylogenetic Analysis of SabHLH Proteins

SaMYC1 and SaMYC4 were grouped with members of the *Arabidopsis* IVa subfamily, SaMYC3 and SaMYC5 were grouped with members of the *Arabidopsis* III d + e subfamily, SabHLH1 was grouped with members of the *Arabidopsis* III b + f subfamily, and SabHLH2 was grouped with members of the *Arabidopsis* X subfamily, while SabHLH3 was grouped with members of the *Arabidopsis* XI subfamily, and SabHLH4 was grouped with members of the *Arabidopsis* IV b + c subfamily (Figure 1d).

### 3.5. Subcellular Localization Analysis

**Figure 2:**
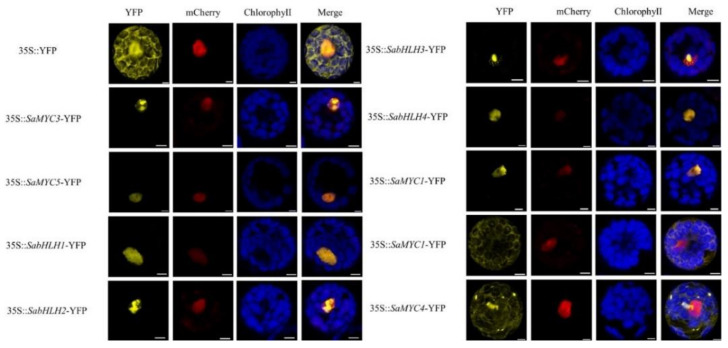
Subcellular location of SabHLHs in *Santalum album*. Note: 35S::YFP was the localization of unloaded vector, yellow fluorescence was YFP fluorescence (indicating protein localization), red fluorescence indicated nuclear-localized protein, blue fluorescence indicated chloroplast autofluorescence, orange with blue fluorescence was a merged image. Scale bars = 5 μm.

A yellow fluorescent protein signal was observed under a laser confocal microscope. In the control group, fluorescence was expressed throughout the entire cell. The fused protein fluorescence signals of SaMYC3, SaMYC5, SabHLH1, SabHLH2, SabHLH3, SabHLH4 and YFP were detected in the nucleus, matching well with the fluorescence signal of mCherry protein located in the nucleus (Figure 2). This indicated that the six TFs were localized in the nucleus. However, SaMYC1 was localized in both the nucleus and the cytoplasm (Figure 2). Finally, the SaMYC4 YFP fluorescence signal was evenly distributed in the cytoplasm, indicating that it was localized in the cytoplasm (Figure 2).

### 3.6. SaMYC1 Conservative Domain Prediction

**Figure 3:**
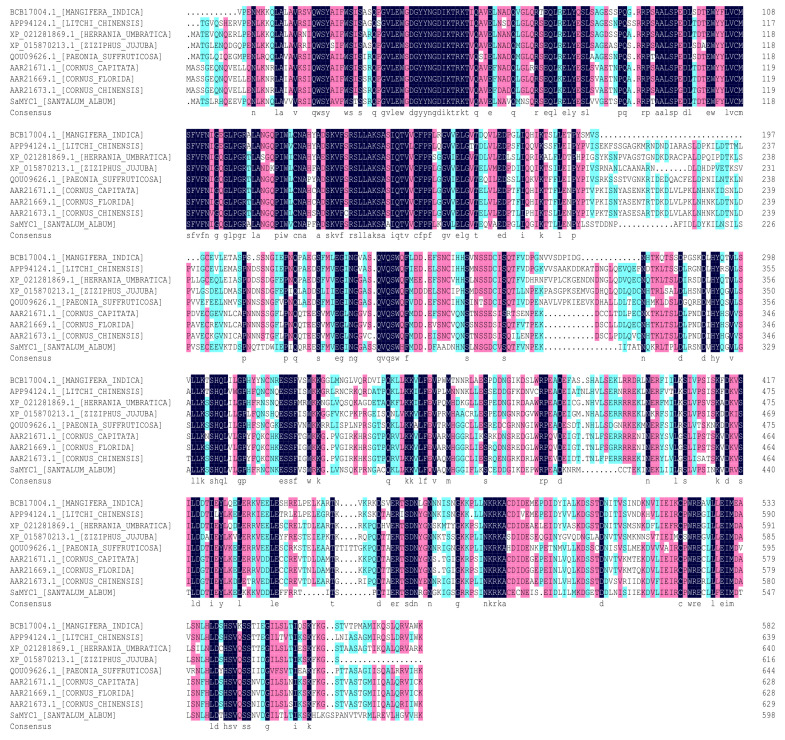
Similarity analysis of the amino-acid sequence of SaMYC1.

It was shown that *SaMYC1* had a typical bHLH-MYC_N superfamily domain at the position of 15–196, and a typical basic-helix-loop-helix domain at the position of 417–460 (Figure 3). 

### 3.7. Analysis of G-Box Elements and E-Box Elements in Promoters SaSSy and SaCYP736A167

The *SaSSy* promoter region had no E-box element; it only contained two identical CAEs, the G-box (CACGTT), which could bind to the bHLH TF, and both were located on the antisense chain (Supplementary Figure S2a). There were six CAEs in the *SaCYP736A167* promoter region that could bind to the bHLH TF. There was no E-box element, but there were only three types of G-box elements, namely CACGTT, CACGTG, and CACGTA, two of which were on the antisense chain and four of which were on the sense chain (Supplementary Figure S2b).

### 3.8. Verification of the Activity of Transcription Factors

The results showed that the control group grew in a well growth status on the AbA-free SD/Leu plates; it was indicated that the yeast strain used in the experiment was intact and active. *SaMYC1* and *SabHLH1* were growing well on SD/-Leu/AbA 0 ng/mL plates and not on SD/-Leu/AbA 200 ng/mL plates, indicating that these two transcription factors were active in yeast and had no self-activation, which could be used for subsequent experiments. *SaMYC4*, *SaMYC5*, *SabHLH3* were not growing well in SD/-Leu/AbA 0 ng/mL plates and should not be used for subsequent yeast single-hybridization experiments. *SaMYC3*, *SabHLH2*, and *SabHLH4* were not growing well at all on SD/-Leu/AbA 0 ng/mL plates, indicating that they may not be active in yeast and cannot be used for subsequent experiments (Supplementary Figure S2).

### 3.9. Screening AbA Concentrations That Inhibiting the Growth of Bait Strains

The bait strains pAbAi-SSy1G-box, pAbAi-CYP1G- box, pAbAi-CYP2G-box, pAbAi-CYP3G-box, pAbAi-CYP4G-box were obtained in *E. coli*. The bait strains p53-AbAi, pAbAi-SSy1G-box, pAbAi-CYP1G-box, pAbAi-CYP2G-box, pAbAi-CYP3G-box, and pAbAi-CYP4G-box grew well in the presence or absence of AbA. However, the growth of pAbAi-SSy1G-box and pAbAi-CYP1G-box strains were completely inhibited, and the minimum AbA concentration to screen them was 100 ng/mL. pAbAi-CYP2G-box, pAbAi-CYP3G-box and pAbAi-CYP4G-box were all able to grow normally at 1000 ng/mL of AbA, indicating that endogenous TFs in yeast had been binded to these bait strains, so they could not be used for subsequent experiments. The growth of the positive control (p53-AbAi strain) was completely inhibited as AbA concentration was 200 ng/mL, indicating that was the minimum AbA screening concentration, so the yeast one-hybridization system was feasible (Supplementary Figure S3).

### 3.10. Assessment of the AbA Concentration That Inhibits the Growth of G-Box Mutant Element Bait Strains

The growth of the bait strain pAbAi-mSSy1G-box was completely inhibited as AbA concentration was 1000 ng/mL. This indicated that the screening concentration could be used in following experiments to preserve the mutant bait strain with a final concentration of 30% glycerol (Supplementary Figure S4).

### 3.11. Detection of the Interaction between SaMYC1 and Mutant G-Box Elements

**Figure 4:**
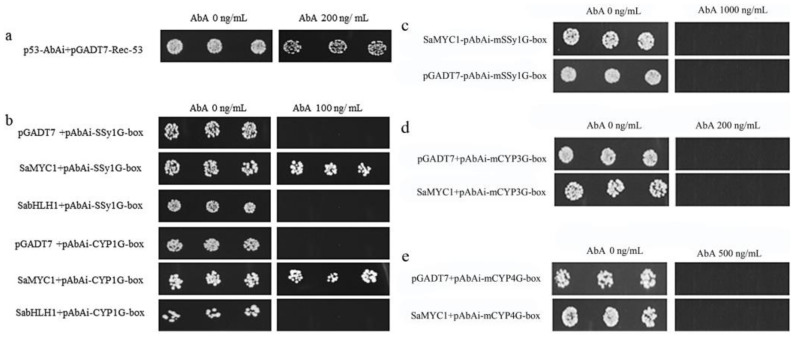
Interaction between SaMYC1 and G-box. Note: (**a**) Positive control; (**b**) Empty vector pGADT7 + pAbAi-SSy1G-box, pGADT7 + pAbAi-CYP1G-box was the negative control. (**c**) empty vector pGADT7 + pAbAi-mSSy1G-box was negative control; (**d**) empty vector pGADT7 + pAbAi-mCYP3G-box was negative control; (**e**) pGADT7 + pAbAi-mCYP3G-box was negative control; AbA concentrations at 0 ng/mL, 100 ng/mL, 200 ng/mL, 500 ng/mL, 1000 ng/mL, respectively, were the SD/-Leu screening media.

The positive control p53-AbAi + pGADT7-Rec-53 could grow well on SD/-Leu medium supplemented with 200 ng/mL AbA. However, *SaMYC1* + pAbAi-mSSy1G-box and the negative control pGADT7 + pAbAi-mSSy1G-box could not grow on the screening medium with 1000 ng/mL AbA, *SaMYC1* + pAbAi-mCYP1G-box and the negative control pGADT7 + pAbAi-mCYP3G-box could not grow on the screening medium with 200 ng/mL AbA, while *SaMYC1* + pAbAi-mCYP4G-box and the negative control pGADT7 + pAbAi-mCYP4G-box could not grow on the screening medium supplemented with 500 ng/mL AbA (Figure 4c–e). This showed that *SaMYC1* could not bind to the mutant elements *mSSy1G*, *mCYP3G*, and *mCYP4G*, and it also illustratrd that *SaMYC1* could combine the G-box element in *SaSSy* and *SaCYP736A167* promoter (Figure 4b).

### 3.12. SaMYC1 Activated SaSSy and SaCYP736A167 Promoter Activity

**Figure 5:**
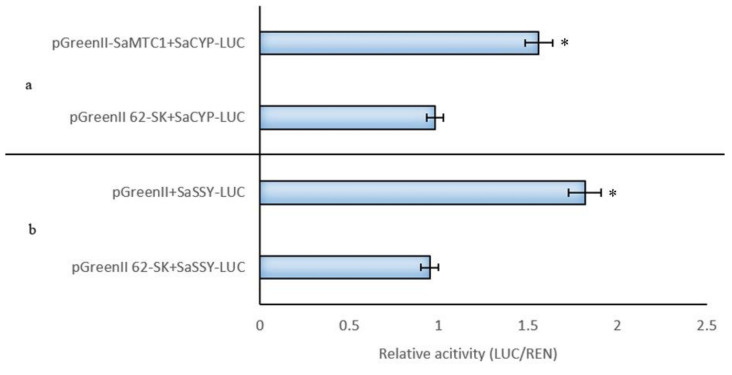
Dual-Luc test verified that *SaMYC1* activated the transcription of *SaSSy* and *SaCYP736A167*. Note: (**a**): Dual-Luc test verified that *SaMYC1* promoted the transcription of *SaCYP736A167*; (**b**): Dual-Luc test verified that *SaMYC1* promoted the transcription *SaSSy* in *Santalum album*. * indicated significant differences using *t*-test (* *p* < 0.05).

As *SaMYC1* was bound to the *SaSSy* promoter, the ratio of *LUC/REN* was 1.85-fold higher than that of the control group, indicating that *SaMYC1* may bind to the *SaSSy* promoter and activate it (Figure 5b). When *SaMYC1* was bound to the *SaCYP736A167* promoter, the *LUC/REN* ratio was 1.55-fold higher than that of the control group, indicating that *SaMYC1* may also bind to the *SaCYP736A167* promoter and activate it (Figure 5a). These findings indicated that *SaMYC1* had activating effects on *SaSSy* and the *SaCYP736A167* promoters. 

## 4. Discussion

bHLH TFs play integral roles in the resistance of a plant to environmental stress. *PebHLH35*, a bHLH TF gene, which localized in the nucleus of *Populus diversifolia*, was induced by drought stress and ABA, while overexpression of *PebHLH35* significantly improved drought tolerance [40]. Since overexpression of the bHLH TF gene *FtbHLH2* in *Fagopyrum* *tataricum* increased cold resistance in *A. thaliana*, it was suggested that this TF played a positive regulatory role in the resistance of *F. tataricum* to cold [41]. Overexpression of the *TabHLH39* gene in wheat significantly enhanced tolerance to salt stress in *A. thaliana* seedlings [42].

bHLH TFs were involved in regulating the synthesis of anthocyanins in *Triticum aestivum* [43]. In *A. thaliana* seedlings, bHLH TFs were involved in the anthocyanin biosynthetic pathway by forming TTG1/bHLH/MYB complexes with MYB TFs and WD40 proteins [44]. In *A. thaliana*, mutations in *MYC2*, *MYC3*, and *MYC4* downregulated gene expression involved in the regulation of glucosinolate biosynthesis [45]. In *Catharanthus roseus*, *CrMYC2* regulated the synthesis of alkaloids by the TF ORCA3, which contained AP2/ERF domains [46]. Overexpression of the *AabHLH1* gene localized in the nucleus of *Artemisia annua* upregulated the expression of structural genes, thereby increasing the accumulation of artemisinin [47]. In *A. thaliana*, *AtMYC2* was bound directly to the promoters of sesquiterpene synthetase genes *TPS21* and *TPS11*, activating their transcription. GA_3_ and jasmonic acid signals could also integrate into the transcriptional regulation of sesquiterpene synthases, and regulate the synthesis of sesquiterpenes [48]. In *S. lycopersicum*, *SlMYC1* differentially regulated the biosynthesis of mono- and sesquiterpenes in the trichomes of leaves and stems, reversed regulation of sesquiterpene synthesis, and caused forward regulation of monoterpenoid synthesis [49]. In the woody plant *Aquilaria sinensis*, bHLH TFs were involved in regulating the synthesis of plant sesquiterpenes: one bHLH TF gene *AsMYC2* upregulated the expression of the sesquiterpene synthase gene *ASS1* in epidermal cells and the expression of *TPS21* and *TPS11* in *A. thaliana* [50].

bHLH TFs play a very important role in regulating the synthesis of plant sesquiterpenes, such as the synthesis of linalool in *Freesia hybrida* [51], linalool and β-caryophyllene in *Chimonanthus praecox* [52], and sesquiterpenes in *S. lycopersicum* [49]. In this study, based on the existing transcriptome data of our research group, the expression patterns of eight bHLH TFs were similar to those of two structural genes, *SaSSy* and *SaCYP736A167*, as screened by co-expression patterns. The eight TF genes were successfully cloned from a mixture of cDNA from the stems and leaves of *S. album*. This was used to construct a phylogenetic tree of these TFs together with members of the bHLH TF family in *A. thaliana*. The eight *S. album* TFs were mainly clustered into six subfamilies (Figure 1d), indicating that these TFs may perform different functions in sandal trees. TFs that were involved in the expression of regulatory structural genes generally bind to the promoter region of a structural gene, and this process typically took place in the nucleus. The subcellular localization results showed that SaMYC3, SaMYC5, SabHLH1, SabHLH2, SabHLH3, and SabHLH4 were all localized in the nucleus (Figure 2), consistent with previous reports in *F. hybrida* and *C. praecox* [51,52], indicating that they have typical characteristics of TFs. However, SaMYC1 was localized in both the nucleus and the cytoplasm while SaMYC4 was localized in the cytoplasm (Figure 2). The phylogenetic analysis indicated that SaMYC1, SaMYC4 and AtbHLH12 clustered in the IV a family (Figure 1d). Therefore, SaMYC1 and SaMYC4 may be able to modify the localization of other TFs such as AtMYC1, or the expression of genes that regulated structures in cells by binding to other TFs. However, this required further experimental verification.

At present, research on the molecular aspects of sandal oil biosynthesis has mainly focused on structural genes, with fewer studies on the transcriptional regulation of genes. In some plants, bHLH TFs played important roles in sesquiterpene biosynthesis [53]. To date, however, there were no reports of the involvement of bHLH TFs in the regulation of santalol biosynthesis in *S. album*. *SaSSy* and *SaCYP736A167* were two key genes-encoding enzymes that functioned downstream of the biosynthetic pathway of santalol sesquiterpenes. The sesquiterpene synthase encoded by *SaSSy* could ligate the substrate FPP into sesquiterpenes unique to santalol, and sandal sesquiterpenes could be oxidized to terpene under the action of oxidase encoded by the *SaCYP736A167* gene. Studies have shown that transcriptional regulation had amplification effects on structural gene functions. At present, in other plants, transcription factors regulated the synthesis of plant sesquiterpenes. For example, in cotton, *GaWRKY1* activated the CAD1-A promoter, thereby promoting the synthesis of sesquiterpenes [54]. In agarwood, the transcription factors *MYB4*, *WRKY4*, *MPKK2*, and *MAPK2* positively regulated the expression of the sesquiterpene synthase gene *ASS1-ASS3*, thereby promoting the synthesis of sesquiterpenes [50]. In *Artemisia annua*, overexpression of *AaWRKY1* activates the expression of the key enzyme gene *AaCYP71AV1*, thereby promoting the synthesis of artemisinin [55]. There were also many studies on the transcriptional regulation of bHLH transcription factors on the synthesis of sesquiterpenes, e.g., in *Artemisia annua*, overexpression of *AaMICC2* transcription factors could improve the transcription level of *AaCYP71AV1* and *DBR2* genes [56], and in Malus pumila calli, overexpression of *MdMYC2* and *MdERF3* could significantly increase the transcription levels of *MdHMGR2* and *MdAFS*, thereby increasing the synthesis of α-farneene [57]. However, it was unclear whether bHLH transcription factors in sandalwood were involved in transcriptional regulation of sandal sesquiterpene biosynthesis. Therefore, it is necessary to study the transcriptional regulation effect of bHLH transcription factors on *SaSSy* and *SaCYP736A167* promoters.

At this stage of this study, the effect of bHLH transcription factors on *SaSSy* and *SaCYP736A167* promoters was mainly explored by combining yeast one-hybridization experiments and dual luciferase experiments. In yeast one-hybridization experiments, *SaMYC1* was determined by screening the medium by transferring *SaMYC1* to two structural gene promoters by transferring *SaMYC1* into bait strains containing G-box elements SSy1G-box, CYP1G-box, and mutant G-box elements mSY1G-box, mCYP3G-box, and mCYP4G-box. The results showed that *SaMYC1* could be combined with the G-box components in *SaSSy* and *SaCYP736A167*. In the dual luciferase experiment, we have constructed the full length of the *SaSSy*, *SaCYP736A167* promoter and *SaMYC1* transcription factor into the reporter and effector carrier of the double luciferase, co-transformed the tobacco in an agrobacterium-mediated manner, and explored the effect of the transcription factor on the promoter of the two structural genes by detecting the chemiluminescence value of the protein of interest. The results showed that *SaMYC1* can activate *SaSSy* and *SaCYP736A167* promoters. Similar to *AtTT8* in *Arabidopsis* that could upregulate the structural genes *DFR* and *BAN* of flavonoid synthesis pathways to promote the biosynthesis of flavonoids in *Arabidopsis* *siliques* [58], *AtGL3* could upregulate the expression of *DFR*, a key structural gene for anthocyanin synthesis [59]. At the same time, many studies have shown that bHLH transcription factors often formed complexes with other transcription factors and were involved in the regulation of transcriptional expression of structural genes. In *Arabidopsis*, bHLH formed complexes with *MYB* and *WD40* subunits to regulate the expression of structural genes along the synthesis pathways of anthocyanins and flavonoids, as well as stamens development and seed formation [59,60,61].

In this study, yeast single-hybridization experiments were tested to find that *SaMYC1* could bind to the G-box element in the promoter of the santalol biosynthetic key enzyme gene *SaSSy*. Double luciferase experiments were used to show that *SaMYC1* could activate the *SaSSy* promoter, it was speculated that *SaMYC1* was a positive regulator of the key enzyme gene in santalol biosynthetic pathway. Results from *A. thaliana* [61], *Vitis vinifera* L. [62], and *Aquilaria sinensis* Lour. [50] indicated that bHLH transcription factors often co-regulated the expression of structural genes in conjunction with other transcription factors. Therefore, our study could also lay a foundation for subsequent exploration of whether *SaMYC1* regulated the expression of *SaSSy* with other transcription factors and how to co-regulate *SaSSy*.

## 5. Conclusions

bHLH TF genes with similar expression patterns and high expression levels were screened by co-expression analysis. All eight TFs had highly conserved bHLH domains and *SabHLH1*, *SabHLH2*, *SabHLH3*, and *SabHLH4*, had highly conserved MYC domains. It was indicated that the eight genes belonged to six subfamilies of the bHLH TF family. Among them, SaMYC1 was found in both the nucleus and the cytoplasm, while SaMYC4 was only localized in the cytoplasm. The remaining six TFs were localized in nucleus. *SaMYC1* could bind to the G-box of *SaSSy* and the *SaCYP736A167* promoter and the *LUC/REN* value was 1.85- or 1.55-fold higher, respectively, than that of the control group. It was inferred that *SaMYC1* could activate both *SaSSy* and *SaCYP736A167* promoters.

## Figures and Tables

**Table 1:** Analysis of physicochemical properties of SabHLH proteins in *Santalum album*.

Gene Name	ORF Length (bp)	Amino Acid Length (aa)	Mw (kDa)	pI	Instability Index	Grand Average of Hydropathicity
*SaMYC1*	1800	599	67.32	5.69	52.17	−0.358
*SaMYC3*	2022	673	72.92	6.12	55.76	−0.533
*SaMYC4*	2151	716	77.72	5.12	64.21	−0.558
*SaMYC5*	1542	513	55.71	5.69	48.26	−0.404
*SabHLH1*	999	332	37.11	4.67	62.30	−0.456
*SabHLH2*	1302	433	47.54	6.09	46.59	−0.700
*SabHLH3*	927	308	32.09	5.91	51.81	−0.374
*SabHLH4*	708	235	25.87	7.71	51.73	−0.766

## Data Availability

All data generated or analyzed during this study are included in this published article.

## Supplementary Materials

The following supporting information can be downloaded at: https://www.mdpi.com/article/10.3390/life12071017/s1. Supplementary Table S1: NCBI login numbers for transcription factors in *Santalum album*; Supplementary Table S2: Premier information in *Santalum album*; Supplementary Table S3: The cluster multiple sequence alignment of SabHLHs in *Santalum album*; Supplementary Table S4: Yeast one-hybrid experimental genes synthesis sequences in *Santalum album*. Supplementary Figure S1: TF open reading frames (ORFs) were amplified by RT-PCR and then electrophoresed on a 0.1% agarose gel. Supplementary Figure S2: Detection of G-box elements of SaSSy ((a) G-box marked blue) and SaCYP736A167 ((b) G-box marked pink) promoter. Supplementary Figure S3: Transcription factor activity verification. Supplementary Figure S4: Screening of the inhibitory AbA concentrations of bait strain. Note: p53-AbAi was the positive control; pAbAi-SSy1G-box was the G-box element strain of SaSSy; pAbAi-CYP1G-box was the G-box element strain of SaCYP736A67; the SD/-Leu selection media with AbA concentration of 0 ng/mL, 50 ng/mL, 100 ng/mL, 200 ng/mL, respectively. Supplementary Figure S5: Screening AbA concentration of mutant G-box element bait strain. (a): Screening of the lowest inhibitory AbA concentration of mutant bait strain pAbAi-mSSy1G-box; (b): Screening of the lowest inhibitory AbA concentration of mutant bait strain CYP3G-box; (c): Screening of the lowest inhibitory AbA concentration of mutant bait strain pAbAi-mCYP3G-box. A-L: The SD/-Leu selection media with AbA concentration of 0 ng/mL, 50 ng/mL, 100 ng/mL, 200 ng/mL, 300 ng/mL, 400 ng/mL, 500 ng/mL, 600 ng/mL, 700 ng/mL, 800 ng/mL, 900 ng/mL, 1000 ng/mL, respectively.
