# Supplementary material for: Identification and Functional Analysis of SabHLHs in Santalum album L
Source: Life (Basel). 2022 Jul 8;12(7):1017. doi: 10.3390/life12071017 (PMC9315531; doi:10.3390/life12071017)
Supplement: Supplementary file 1 [file life-12-01017-s001.zip › life-1746853-supplementary.pdf]

Supplementary Tables and Figures

Supplementary Table:

Table S1. NCBI login numbers for transcription factors in *Santalum album*

Genes	NCBI login numbers
SaMYC1	MZ297933
SaMYC3	MZ297935
SaMYC4	MZ297937
SaMYC5	MZ297938
SabHLH1	MZ297934
SabHLH2	MZ297936
SabHLH3	MZ297939
SabHLH4	MZ297940

Table S2. Premier information in *Santalum album*

Premier names	Premier lists（5′→3′）	Usages
SaMYC1-ORF-F	ATGGCTACTTCACTCCGACATCA	ORF
SaMYC1-ORF-R	TCAGAACTTGTGGACAACACCAT	ORF
SaMYC3-ORF-F	ATGAACCTGTGGAGTGACGACAA	ORF
SaMYC3-ORF-R	CTACCGGGTTTCCGATAATTTGGAA	ORF
SaMYC4-ORF-F	ATGGCTTCGCTGCCGCCACCGCC	ORF
SaMYC4-ORF-R	TCATTCATGCGGAATTATTTGGTTT	ORF
SaMYC5-ORF-F	ATGGAAGATGTGCTTGTCTCCTCCT	ORF
SaMYC5-ORF-R	TTACTCTTCAAATCGTTTAATAAGA	ORF
SabHLH1-ORF-F	ATGGAGTTCAACAATGAGCATGGCT	ORF
SabHLH1-ORF-R	CTACAGGCACCTCCCGCCATACCCT	ORF
SabHLH2-ORF-F	ATGGGAGAGTTAAGCACCGAGAGCA	ORF
SabHLH2-ORF-R	TCATCTATACAAGCATCCTCTGTAA	ORF
SabHLH3-ORF-F	ATGGCGAATAACCCTTCTGAAGGT	ORF
SabHLH3-ORF-R	CTACGGAGGAGGATTAGTTTCGGGCT	ORF
SabHLH4-ORF-F	ATGCTGTCCCAGGATAACACCAATT	ORF
SabHLH4-ORF-R	TTAGGCAACGGGGGGACGGAGTACA	ORF
YFP-SaMYC1-F	CTCAAGCTTCGAATTCATGGCTACTTCACTCCGACATCAG	SaMYC1-YFP vector
YFP-SaMYC1-R	CTCACCATCAGGATCCTGAACTTGTGGACAACACCATGA	SaMYC1-YFP vector
YFP-SaMYC3-F	CTCAAGCTTCGAATTCATGAACCTGTGGAGTGACGACA	SaMYC3-YFP vector
YFP-SaMYC3-R	CTCACCATCAGGATCCTCCGGGTTTCCGATAATTTGGA	SaMYC3-YFP vector
YFP-SaMYC4-F	CTCAAGCTTCGAATTCATGGCTTCGCTGCCGCCACCGCC	SaMYC4-YFP vector
YFP-SaMYC4-R	CTCACCATCAGGATCCTTTCATGCGGAATTATTTGGTTT	SaMYC4-YFP vector
YFP-SaMYC5-F	CTCAAGCTTCGAATTCATGGAAGATGTGCTTGTCTCCTCCT	SaMYC5-YFP vector
YFP-SaMYC5-R	CTCACCATCAGGATCCTCTCTTCAAATCGTTTAATAAGA	SaMYC5-YFP vector
YFP-SabHLH1-F	CTCAAGCTTCGAATTCATGGAGTTCAACAATGAGCATGGC	SabHLH2-YFP vector
YFP-SabHLH1-R	CTCACCATCAGGATCCTCAGGCACCTCCCGCCATAC	SabHLH2-YFP vector
YFP-SabHLH2-F	CTCAAGCTTCGAATTCATGGGAGAGTTAAGCACCGAGA	SabHLH2-YFP vector
YFP-SabHLH2-R	CTCACCATCAGGATCCTTCTATACAAGCATCCTCTGTAAG	SabHLH2-YFP vector
YFP-SabHLH3-F	CTCAAGCTTCGAATTCATGGCGAATAACCCTTCTGAAG	SabHLH3-YFP vector
YFP-SabHLH3-R	CTCACCATCAGGATCCTCGGAGGAGGATTAGTTTCG	SabHLH3-YFP vector
YFP-SabHLH4-F	CTCAAGCTTCGAATTCATGCTGTCCCAGGATAACACCAAT	SabHLH4-YFP vector
YFP-SabHLH4-R	CTCACCATCAGGATCCTGGCAACGGGGGGACG	SabHLH4-YFP vector
62S-SaMYC1-F	TCTAGAACTAGTGGATCCATGGCTACTTCACTCCGACATCA	SaMYC1-62-SK vector

62S-SaMYC1-R	GACGGTATCGATAAGCTTTCAGAACTTGTGGACAACACCAT	SaMYC1-62-SK vector
62S-SaMYC3-F	TCTAGAACTAGTGGATCCATGAACCTGTGGAGTGACGACAA	SaMYC3-62-SK vector
62S-SaMYC3-R	GACGGTATCGATAAGCTTCTACCGGGTTTCCGATAATTTGGAA	SaMYC3-62-SK vector
62S-SaMYC4-F	TCTAGAACTAGTGGATCCATGGCTTCGCTGCCGCCACCGCC	SaMYC4-62-SK vector
62S-SaMYC4-R	GACGGTATCGATAAGCTTTCATTCATGCGGAATTATTTGGTTT	SaMYC4-62-SK vector
62S-SaMYC5-F	TCTAGAACTAGTGGATCCATGGAAGATGTGCTTGTCTCCTCCT	SaMYC5-62-SK vector
62S-SaMYC5-R	GACGGTATCGATAAGCTTTTACTCTTCAAATCGTTTAATAAGA	SaMYC5-62-SK vector
62S-SabHLH1-F	TCTAGAACTAGTGGATCCATGGAGTTCAACAATGAGCATGGCT	SabHLH1-62-SK vector
62S-SabHLH1-R	GACGGTATCGATAAGCTTCTACAGGCACCTCCCGCCATACCCT	SabHLH1-62-SK vector
62S-SabHLH2-F	TCTAGAACTAGTGGATCCATGGGAGAGTTAAGCACCGAGAGCA	SabHLH2-62-SK vector
62S-SabHLH2-R	GACGGTATCGATAAGCTTTCATCTATACAAGCATCCTCTGTAA	SabHLH2-62-SK vector
62S-SabHLH3-F	TCTAGAACTAGTGGATCCATGGCGAATAACCCTTCTGAAGGT	SabHLH3-62-SK vector
62S-SabHLH3-R	GACGGTATCGATAAGCTTCTACGGAGGAGGATTAGTTTCGGGC T	SabHLH3-62-SK vector
62S-SabHLH4-F	TCTAGAACTAGTGGATCCATGCTGTCCCAGGATAACACCAATT	SabHLH4-62-SK vector
62S-SabHLH4-R	GACGGTATCGATAAGCTTTTAGGCAACGGGGGGACGGAGTACA	SabHLH4-62-SK vector
GAD-SaMYC1-F	GAGGCCAGTGAATTCATGGCTACTTCACTCCGACATCAG	SaMYC1-pGADT7 vector
GAD-SaMYC1-R	GAGCTCGATGGATCCCGAACTTGTGGACAACACCATGAAG	SaMYC1-pGADT7 vector
GAD-SaMYC3-F	GGAGGCCAGTGAATTCATGAACCTGTGGAGTGACGA	SaMYC3-pGADT7 vector
GAD-SaMYC3-R	CGAGCTCGATGGATCCCCCGGGTTTCCGATAATTTGG	SaMYC3-pGADT7 vector
GAD-SaMYC4-F	GGAGGCCAGTGAATTCATGGCTTCGCTGCCGCCACCGCC	SaMYC4-pGADT7 vector
GAD-SaMYC4-R	CGAGCTCGATGGATCCCTTCATGCGGAATTATTTGGTTT	SaMYC4-pGADT7 vector
GAD-SaMYC5-F	GGAGGCCAGTGAATTCATGGAAGATGTGCTTGTCTCCTCCT	SaMYC5-pGADT7 vector
GAD-SaMYC5-R	CGAGCTCGATGGATCCCCTCTTCAAATCGTTTAATAAGA	SaMYC5-pGADT7 vector
GAD-SabHLH1-F	GAGGCCAGTGAATTCATGGAGTTCAACAATGAGCATGG	SabHLH1-pGADT7 vector
GAD-SabHLH1-R	GAGCTCGATGGATCCCCAGGCACCTCCCGCCATACCCTG	SabHLH1-pGADT7 vector
GAD-SabHLH2-F	GGAGGCCAGTGAATTCATGGGAGAGTTAAGCACCGAGA	SabHLH2-pGADT7 vector
GAD-SabHLH2-R	CGAGCTCGATGTTCTATACAAGCATCCTCTGTAAG	SabHLH2-pGADT7 vector
GAD-SabHLH3-F	GGAGGCCAGTGAATTCATGGCGAATAACCCTTCTGAA	SabHLH3-pGADT7 vector
GAD-SabHLH3-R	CGAGCTCGATGGATCCCCGGAGGAGGATTAGTTTCGG	SabHLH3-pGADT7 vector
GAD-SabHLH4-F	GGAGGCCAGTGAATTCATGCTGTCCCAGGATAACACCAA	SabHLH4-pGADT7 vector
GAD-SabHLH4-R	CGAGCTCGATGGATCCCGGCAACGGGGGGACG	SabHLH4-pGADT7 vector

Table S3. The cluster multiple sequence alignment of SabHLHs in *Santalum album*

1. SaMYC1 1800kb g11854.t1
ATGGCTACTTCACTCCGACATCAGGAGGAAGTGCCACAAAACCTAAAAAATCAGCTTGCTGTTGTAGTTAGAAGCATTCAATGGAGCT<br>ATGCAGTCTTCTGGTCCACTTCAAGTAGACAACCAGGGGTGTTGGAATGGGGAGATGGGTACTACAATGGGGATATTAAGACGAGGAA<br>AACAGTTCAGGCTGTGGAGCTTAACGCGGTCCAAATGAATTCGCAGCGAAGCGAGCAGTTGAGAGAACTCTACGAGTCCCTTGTTGTG<br>GGTGAAACCAGCCCACAAGCTCGGAGGCCAACGGCTGCATTGTCCCCTGAAGACCTAACCGATACTGAATGGTTTTACTTGGTTTGCAT<br>GTCCTTTGTATTTAACATTGGCCAAGGTTTGCCGGGAAGAGCATTGGCCAATGGTCAACCCATCTGGGTCTGTAACGCTCCCTTTGCAGA<br>TAGTAAAGTATTCAGCCGTTCTCTGCTAGCTAAGAGTGCCGCTATTCAGACTGTTGTATGCTTTCCATTTTTAAAAGGAGTGCTGGAGCT<br>GGGTGTGACTGAGCAAGTCGCGGAGGATCCTGGTCTCATTCAGGGCATAAAAACTACTTTGTTGGAGAATCCATACCTCTCAAGTACTG<br>ATGACAATCCAGCTTTCATTGACCTGGATTATAAGATTTTGAACAGCATTCTTAGCCCAGTTTCTGAATGCGAGGAAGTCAAAACTGATT<br>CTCCTAATCAGACCACAGATTGGATTGAACCCATTCAACAAAGAGAGGAGTCTTTTATGGTTGAAGGGATCAATGGGGGTGCTTCTTCT<br>CAAGTACAAAGTTGGCATTTTATGGACGATGATTTCGCTGCTGATAACCATAATTCCCTGAACTCTGGCGATTGTGTATCTCAAACCTTT<br>GTAAATCCTGAAAAAATTATCACTGCTACAAATCAGAAGAGACTGACCCCACTGGATCTTCGGAGCAATGACGTACATTATCAGGGAG<br>TTCTTGGAGCCCTTCTGAAGAGTTCTCACCAATTGGTCTTAGGACCACACTTCAGAAATTGTAACAAGGAGTCTAGTTTTGTTAGTTGGA<br>GAAAAGGATTGGTAAATTCCCAGAAGCCGAGAAATGGAGCCTGCCAGAAATTACTGAAGAAGGTATTGTTTCAGGTGGCCCAAATGC<br>ATGGTGGCATTTTCCTGAAGTCCTGTGAAGATGATGGCATTAAGGATGAACCTTGGAGACCAGAGGCTGATAAAAATAGAATGTGTTG<br>CACGGAGAAAATAAACGAAAAGCTTATTATTCTCAGATCGTTGGTCCCCTCGATTAACAAGGTTGACAGGGTTTCTACACTTGATGACA<br>CTATAGAGTACCTGAAAGAGCTTAAGAAAAAGGTTGATGACTTGGAATTTTTTAGAAGAACAATAACATCTCCAGATACCATAGAGAG<br>AACATCAGATAATCGTGGCAATGGCAAAATCGGTAGTGGAAGGAGGCCATCGATAAATAAGAGGAAGGCTTGTGAATGCAATGAGAT<br>TTCTGAGATTGACTTGATTTTAATGAAAGATGGTGAAACAGATGATTTGAATATAAGCATCATTGAGAAGGATGTGCTGATTGAAATCA<br>GGTGCCCATGGAGGGAGTGCTTGTTGTTCGAGATCATGGATACGCTAAGCAATCTACATTTGGATACTCACTCGGTACAATCATCTAAT<br>GTTGATGGAATTCTTACACTGACCATTAAATCTAAGCACTTGAAGGGTTCACCTGCTAATGTAACAGTGAGGATGCTCAGAGAAGTACT<br>TCATGGTGTTGTCCACAAGTTCTGA
**2. SabHLH1 999kb g17775.t1**
ATGGAGTTCAACAATGAGCATGGCTTCTTTGAAGAGTTACTTTCCCTGAGAAGAGACAATGCTAGCTGGGTTGAAACCATCCCCACCAC<br>AGGAATGTCTGAATTCTGCCCCAATCCCAGCAGTTTCGAGGACTTCTACGAATACCCAAATGAGATCACTTCCCTCATTTCTACTTCTCC<br>TTCCCTGGAGTCCTTCTCTGCTCCGGTTCTCGAGCAGGGCCTGTTTGAGTACCCCAGCAGTGAGCTCTGCTGCCCGTTTGGGGACGGTTT<br>CTCGGCGATGGAAGTTACCGATATGCCCCCGTTCCCCGGGCAGGGGGTGGAGGACGATTTCGGGCGGGGTCGAGCCGGGACTGGACCG<br>GAGGGGGGCTTGGAGATGTTGGACGCATGTAAAGTGGAGCCGGGTCAGGGATCCGAAACCGCGGTTTTCGACATCGGGTTCTGCCTCG<br>ATCGGAAGAGCAACCGAGGGAAGAAGGTGGAGGGGCAGCAGTCGAAGAATCTTATGGCGGAGAGGCGGCGGAGGAAGCGGCTGAAT<br>GATCGGCTCTCGATGCTCCGATCCGTCGTTCCAAAAATAAGTAAGATGGACAGGACATCTATCCTTGGGGACACCATAGATTACATGA<br>AGGAGTTGCTAGAGAAAATCAACAATCTGCAAGAAGAATCAGATGTGGACTCCAAGCAGCTGGACTTGATGGGTATATTCAAGGACA<br>TAAAGCCCAATGAAATTCTAGTCAGAAACTCACCCAAGTTCGATGCGGAAAGGAGGAACTCGGATACCCGGATCGAGATTTGCTGCAC<br>CGGGAAGCCTGGATTGTTGCTGTCTACTATGACGACGCTCGAGGCATTAGGCCTTGAAATCGAGCAGTGTGTCATAAGCTGCTTTAACG<br>ATTTTGCGCTCCATGCTTCGTGCTCAGAGGATTCGGAGCAAAGAACAGTAATAAGCTGTGAAGACATAAAGCAAGCGTTATTTAGGAA<br>TGCAGGGTATGGCGGGAGGTGCCTGTAG
**3. SaMYC3 2022kb g7080.t1**
ATGAACCTGTGGAGTGACGACAACGCGTCGATGATGGAAGCTTTCATGAGCTCTGAACTCTCCTCCTACGGGTGGGCCCCTCAGTCCTC<br>CTCGTCCGTCGCCTCCACCTCCCACCCCGCTCCTCCTCCTCCTCCTCCTCCGCTACCGACGGCTCCTCCTCCGACCACCGCCGGCGTGGG<br>ACAGTCTCAAGCTCCTCCCATGGCGTTCTTCAACCAGGATACCTTGCAGCAGCGGCTCCAGACCCTTATCGAGGGCGCCCGCGAGAGCT<br>GGACCTACGCCATCTTCTGGCAGTCCTCGGTTGATTTCTCCGGCGCCGCCCTGTTGGGGTGGGGCGATGGCTACTACAAGGGGGAGGAG<br>AACAAGTGCGGTGGCGGGGGGAAGCGCCGAAGGCTAAGCAACCCCGCCACCGCCGCCGAGCAGGAGCACCGCAAGAAGGTCCTCCG<br>CGAGCTCAACTCCCTCATCTCCGGCGCTTCCGCATCGGCTGCCGCCGTCGACGATACCGTCGACGAGGAGGTCACCGACACCGAGTGG<br>TTCTTCCTCGTCTCCATGACGCAGTCCTTCGCCAGCGGGATGGGTCTCCCCGGTCAGGCCTTCTTCACCTCCAACCCCGTCTGGCTCGCC

GGTCCGGAACGCCTCTCCGGTTCGACCTGCGAGCGCGCGCGACAGGGGATGGGCTTCGGCCTTCGGACGATGGTGTGCATCCCGCTGGT
GAACGGGGTCGTCGAACTGGGGTCCACGGAGTTGATTCTCCAGACGCCAGATCTGATGAACAAGGTTAGGGTTCTGTTCAATTTCAGTA
GTTCGGAGATCAGCTCGTGGCCAATTCATGGCGATCAGCGCGAGAACGAACCCCACTCCGCAATCTGGGTTCCCGACCACCCACCTCC
TCCCTCCTCCGCCGTCGAAATTAGGGATTCTGTAAGCGCCGCACCACCACCACCAATCCCCACTCCCAATCACAATTCCAATCCCAGTT
CGAGTACCCTAACCGATCTAAACCCTAACCCTAGTTCTTCAAATGTAAAATCCCACACCTACCCGGCGCGGTTGGTAACGACCCCGGCG
CCGGCGCCACTGCAGACGCAAAGCTTCTTCACGAGGGAGATAAATTTCGCCGAATTCGGGTACGAGGGAAGCGGCGGCGGCGGAGTG
AGGACCGGCGGGTGCAAGCCGGAGTCGGGAGAAATCTTGAATTTCGGGGAGAGTAAGAGGAGCTCGTGTAATGGGAATGGGAGTTTA
TTCTCCGGCCACCCTCAGCTCGTAGCCGAGGAGAATTGTCGGAAAGGGCGGTCTCCGGCGTCGCGAGGTAGCAACGAGGAGGGAATG
CTGTCCTTCTCCGGGATGGTGAAATCCACCGGCGGCGGCGCCGATTCGGACCAGTCCGATCTCGAGGCCTCGGTTGTGGTCAGAGAAAC
TGACAGTAGCAGAGTGGTGGATCCCGAGAAACGCCCCCGGAAACGGGGACGAAAGCCCGCCAACGGGAGGGAGGAGCCGTTGAACC
ACGTCGAGGCGGAGCGGCAGCGGCGCGAGAAGCTTAACCAGAGATTCTACGCGCTCCGCGCCGTCGTCCCCAACGTCTCGAAGATGG
ACAAGGCGTCCCTCCTGGGCGACGCCATCGCGTACATTAACGAGCTCCGCACCAAGGTCCAGACCGCGGTCTCCGAGAAGGAAGAGTT
GCAGAAAGAGGTGGCGGGGTTGAAGAAGGAGAAAGCAGTTCCCTCCCCCGAACAACACCCCCCTAAATCGTCGGACCATGGCGGGGA
GAAGTTGGTGGACATTGAAATCGACGTGAAGATCATCGGTTGGGACGCGATGATCAGAATACAATGTAGTAAGAGGAATCATCCGGC
GGCGAAGTTGATGGCGGCGTTGAAAGAGCTGGATTTGGATGTTCACCATGCGAGCGTTTCTGTAGTGAAAGATTTGATGATACAGCAA
GCAACATTGAAGATGGGGAGCCGATTTACACGCAGGAACAGCTCAAAATGGCCTTATCTTCCAAATTATCGGAAACCCGGTAG

4. SabHLH2 1302kb g31888.t1

ATGGGAGAGTTAAGCACCGAGAGCACTTCTCTTGCTATTTCTTCCTCTTCTTCGGCTCACAACTGGTGGGATCTCCATTCAACCTCATGG
AACCCTAGTGTCCATACCCATAACCCTTGGAACTCTTTGAACCCTAGTTCCAATTCTCCCACTTGTGAGGAGGATGTTTCGATCTCCACA
TCGTTCACCAACGGTTCCAATCACTCCGGCGTGAGCGTCGATTCCTCTCACCAGTTCGGTGAATCCCATGAGTTGATTGGAGAAATCGT
GTCGGATAATCATATGTGGAACCATGTTCTCTTAAGCAGCGGTGGTGGCGATCATGGGATGATGTTCCAGTTACCTCAGAACAATAACT
GGGAGTTCTCTAACACCAATTACTTTGACAAAACGCACCACTTGAATGGATTCATCCACAGCGACATAACAAGCAATGAAAATGAAAG
CAACAATAGTAAGAATCTCTCTAATTTGGTCAGCAATTGGTCCATTGCTCCTCCATGCCCACAAGCCGACCATCTCGATCCCCAACCGT
ACGATAATCAGGGTCTGTTTTTGCACTCCGGCGACACAAAATCGATCCGCAGGGATGACAGCAGTGGTGGTTTATTACAGAGGTTGCAG
GCAGCGGCGCCGTCATTGGATGCGAATTATGGTGCTAGTGAAAGTTGTTACTATGGTGGTGGCCTTGAAGACAATAATAATGGGAGAA
AATTATGTGATGTTATATCATTTAGTGGTAGTAGCAATGGTGTGAAGCCCAGCTGCCAGAAATCCTTGAATTTATCAGCTGGGTGGAGG
AAGCAAGGTCTCTTTACTTCTAATTCCCCAGTGAAAAACAGTGGAAGAAGACAGATCATCAACAATGAAGTAAAGAAGAGAAGATCA
AACAGCAATAGTTCAGAGACGCTCCCCAAAAAGCAAAAGCATGAAAGCAGTGCAGTTTCATCGGTAAAGATCATGGCACCAAAAGTC
AAGCTTGGGGACAAGATCAGTGCTCTTCAGCAAATTGTGTCACCATTTGGAAAGACAGACACAGCGTCTGTGTTATTAGAAGCAATCG
GATACATAAGGTTTCTACAAGAGCAAGTGCAGCTGCTTAGCAATCCTTACATGAAGACTATGCCATGCAAGGATCCATGGGGAGCTCT
GGACAGAAAAGAGCAAGGAGATGCAGAATTGGACCTTCAGAGTAGAGGTCTTTGTTTAGTTCCAACCTCTTGCACCCCTCAAACGTAT
CATGAGAATGGGGTTGGATCAGATTATTGGACACCCACTTACAGAGGATGCTTGTATAGATGA

5. SaMYC4 2151bp g13570.t1

ATGGCTTCGCTGCCGCCACCGCCGCCCGCACTGAGCACTCGATTGCAGTCCATGTTACAGACCGCAGTCCAATCCGTGCAGTGGACTTA
CAGTCTCTTTTGGCAGTTTTGTCCGCAACAAGGGATTCTGGTGTGGGGGGATGGGTACTACAATGGGGCAATAAAGACGAGGAAGACG
GTGCAACCGGTGGAGGTGAGCACGGAGGAGGCGTCGCTGCAGCGGAGCCAGCAACTCCGAGAGCTCTACGACTCGTTATCGGCCGGA
GACACGAACTCGCAGCAGCCGCCTCGCCGCCCCTCCGCCTCGTTGTCGCCGGAGGACTTGACCGAGTCGGAGTGGTTCTATCTCATGTG
TGTCTCCTTCTCTTTTCCTCCCGGCATCGGGTTGCCGGGGAAAGCATATTCAAAGAGGCAGCATGTGTGGCTTACGGGAGCAAACGAGG
TTGACAGCAAAGTGTTTTCTAGAGCTATTCTTGCTAAGAGTGCTCGCATACAGACTGTGATGTGCATTCCTCTATTAGACGGGGTCGTTG
AGCTTGGCACCACAGACAAGGTGCAAGAGGACCACGGATTGATCCGCCGAGTGAAAAGCGTCTTCACGAACCACAGCAATGATCCCC
CTCCTCCCAAGCCGGCCCTCTCCGAGCACTCCACTTCCAATCCCGTCTCCTCTGCCGCCGCCCTCCACCCCACATTGTACGCAGGTGCCG
CAGCAGCAGACCCGGACGGAGAAGAAGAAGAAGAAGGAGACGACGATGACGACGATGATGATGACGACGAAGACGACGGAGGAGG
AGGAGGAGGAGGAGGAGCCGAGTCGGAATCAGAAGCCGGGAACGGGCAGAACAACATGGATTTCTCGGTCAGGCATGTGGAGGCCA
TGGGGGTGGGGACGGCCGAGCCGAGCGAGCTCATGCAGCTCGAGATGTCGGAGGATATTCGGATTGGCTCGCCCGATGACGGGTCGA
ACAACCTCGACTGCGACTTCCACTTGCTCGCCGTGGCTCAGTCGGAGCACCCGACGGCGGAGGCGGATCTTCAGCGCCGGGTTGACTC
GTACAGGGCTGAGTCGAGCCGGAGGTGGCCGACGGTGCCGGAAATGGGTATTACTGCGATGATTAAACCGCCACCTTCAGGTGGAGA

GGGTTTCAATCCACATCCACTAGATGAATTGACCCAAGAGGACACACACTACTCCCAGACTGTCTCCACCATCCTCCAACACCAGTCCG
CGAGGTGGTCCTCCGCCGCCGCCGGGATCCCCTCCATCTACTCCTCCCGCTCCGTTTTCTCGCGGTGGACCTCCCACTCCGGCGACCTCG
CCACCTCCGTCGCCGCCGACGAGACCTCCCAGTGGCTCCTCAAGTACATCCTCTTCAGCGTCCCTTTCCTGCAAGCCAAGTCCCGCGTCC
CCGCCGTCGCCTCAGCCGGAGACCAGGACTCGCCGAAATCCCGCCGGGGCGGCGGAGGAGGCGGGGGGACGCCGCAGGAGGAGCTC
AGCGCGAACCACGTGCTGGCGGAGCGCCGGCGCCGCGAGAAGCTGAACGAGCGGTTCATCACCCTGCGGTCGCTGGTCCCGTTCGTGA
CGAAGATGGACAAGGCGTCGATCCTGGGCGACGCCATCGAGTACGTGAAGCAGCTCCGCCGCAAGATCCAGGACCTGGAGGCTCGCG
CGCCGCAGGCGGACCTCGACCGCCGCTCCCTCAGCAGATCTAGGGATCCGGATCCGGATCCTCAGCGGAGCGGGTCGAGCGCCTCGGT
GGTGGATCGGACGGGTCGTTCTGGTTCCGGGCAGGACAAGAGGAAGGTCAGGGCCGTGGAAGGGAGCGGCGGTAAGCCGAGGCTGGT
CGGCTCTCCTCCGCCGCACCCGGCGGCAGGCGGCGGTGGATCGGAGTTTGTGAGCATAGAGGTGTCGATCATAGAGAGGGATGCGCTG
TTGGAGTTGCAGTGTGGGTACAGAGAAGGGCTGTTGCTCGACATAATGCAGTTGCTGAGGGACCTCCGCGTTGAAACGACGTCGGTTCA
GTCTTCTCTCAACAATGGCGTTTTCGTGGCCGAGTTGAGGGCCAAGGTGAAGGAGAATGCTAATGGGAAGAGAGTGAGCATTGTGGAA
GTGAAGAGAGCGATAAACCAAATAATTCCGCATGAATGA

6. SaMYC5 1542bp g18261.t1

ATGGAAGATGTGCttgtctcctcctcctcttcctctcttttccccaATTCCAACATGTCTCACCAACCATATATGAGTCAACCCATCACCCCCACCGCCGCC
GATTCACCCACTCTGCAACAGCGGCTTCAGTTCATAATCCAAACCCCAGATCCCTCCTCCTGCTGGTGGGACTACGCCATTTTCTGGCAA
TCCTCCAAAGAAGGCAGCAACCCCAACGGCTGCTCCGCCGTGTTAGCGTGCGGCGACGGCCACTTCCAGAGCCTCCCCGCCGGCGCCG
GCACCGCCCGAGTCTCCCGCAAATCCGGGGTCGGCTCCCGGGAGGCGGAGCGGAGGAAGGCGATGAAGGGGATCCAGGCCCTTCTCG
GGTCGAACCCGGATTTCGACGCATCCGTCGATGGCGACGGCGATGGGGACGTCACCGACGTCGAGTGGTTCTACTTCATGTCCTTCACG
CGGCGGTTCTCTGCCGGCGACGGCGGACCCGCCGACGCCCTGGCTTCCGGATCCACAGTGTGGATCACGGGTCGAGAGGAGCTCCAGC
TCTCCAACTGCAACAGAGCCAAAGAAGCCGAGGCCCACGGGTTAGAGACCTTCGCGTGTATCCCCATACCCGCCGCCGGCGGAGTGCT
CGAGTTGGGGTCGGCGGACCTGATCCGCGAGAACTGGGCTCTCGTCCAACAAGCCAAGACCGTGTTCGGGTCGTACATCCCCAACGGC
TTTCAATTCTTCGATCACCATCACCACCGGAGAAACATTTCTTTTCCCGACACCGGAATAATTTCCGGCGACAAGAACCCATTAACAGA
TCCACCGGAAGGGAACAAGAAGGATCAGTCTCCTTGCGACTATTTGGTGGATTCGGAGCATTCGGATTCGGATTATCCGGCAATGATCG
CTGCGCAGACGAGCACGACCACATCCGTGGATGTGGAGAAACTGGTTAGGGTCCCGAAGAAGAGGGGGAGAAAGCCCGGGGTGGGG
CGCGAGGCGGCGCTGAACCACGTCGAGGCGGAGCGGCAGCGGCGCGAGAAGCTCAACCACCGGTTCTACGCGCTGCGGGCGGTGGTC
CCGAACGTGTCCCGGATGGACAAGGCGTCCCTCCTGGCGGACGCCGTGTCCTACATTAGCGAGCTCAAGGCCAAGGTGGAGGACCTGG
AGTCCCAATTGCCTCACCCCGCTAAGAAACCGAAACGAACCGCAACCGAAACCGTCGAAAATCAAAGCACCATCACTTCAACAGCAA
GCGTCGAGCAGGCCGCGAGGGGTGCTGCTGCTACGACGACTAGTAGCGTGGCGCTGGAAGTGGAAGTGAAGATCGTGGGGCCCGAGG
CCATGATCCGAGTCCAGTCGGAGAATGTAAATTCCCCGTCGGCGAGGCTGATGAACGCGCTCCGTGATCTCGAATTCCAGCTGCACCAT
GGGAGCATGTCTTGTGTGAATGACCTCATGCTCCAAGACGTCGTCGTTCAAGTTCCCGGTATGTTAAAAAGTGCCGAGGCGGTCAAGGT
CGCTCTTATTAAACGATTTGAAGAGTAA

7. SabHLH3 927kb g13803.t1

ATGGCGAATAACCCTTCTGAAGGTGCTAGCGATGATTTCCTCGAGCAGATTCTGGGGATTCACTCCTACTCCGGTGCCGATTCCGGCCTG
TCGTCCGCCACTGACGGCGGAGGTGGCATGCCGGGATCCGCCGCTCCGATGATGCTCCAGCTCAGCTCCGGAGACGGTTCCGGCCATG
TTGGGGGCGTCGTTCCCGGCGGAGGCGGCGGCGGCGGGTATCACGGCACGGTGTTCCCGCTGGGGCTGAGCTTGGATCAGGTGAAAAC
GGGTTTCCTGAAGCAGGAGGAAGCTTCTGGAAGCGGTAATCGGTTTAGGGAGGAGTTTGGGGGTGGTGGTGGTGGTGGTAGAGGCTCT
TCCGCGAAAAACTCTTTCCATGGCCAACCAATGCCTACAGCTGCTGCTGTGCCACACCCCCCTGCAATTCGAACTAGGGTGCGGGCAAG
ACGAGGTCAGGCGACGGATCCACACAGCATTGCTGAGCGGTTACGTAGGGAAAGAATAGCAGAAAGAATCAGAGCACTGCAGGAAC
TAGTTCCTAGCGTCAACAAGACTGATAGGGCGGCCATGCTTGATGAAATTGTTGACTATGTGAAGTTTCTAAGGCTCCAAGTAAAGGTT
TTGAGCATGAGTAGATTAGGCGGAGCTGGTGCAGTGGCACCTTTGGTAACCGACATCCCGTTATCGTCAGCTGAGGAAGAAGGTAGTG
AAGGTGGGAGAAGCCAACCAGCATGGGAGAAATGGTCAAATGATGGTACAGAACGACAAGTCGCCAAACTCATGGAAGAAAATGTG
GGGGCTGCAATGCAATTTCTTCAATCCAAGGCACTTTGCATCATGCCAATTTCACTCGCCTCGGCAATTTACCAAACAGCTCAGCCAGA
TAATACAAACATGCTCGTGAAGCCCGAAACTAATCCTCCTCCGTAG

8. SabHLH4 708kb g2451.t1

ATGCTGTCCCAGGATAACACCAATTGGCTGTTCGATTACGGATTGATCGAGGATATCGCTGCACCAGACGGGAATTTCCCTGCTCCGGC
TTCGGGCTTCACCTGGCCATTACAGGCCCGGAATGATCCTCCTTATCTCGGTGTTGATGTCGATGGCTCACTCGGGGATTCAGATTGTCC
CAAAGAAACAGGCTCAAAAAAGAGGGGGAGATCTGAACCATCTGGCCCTTCTAGTTCCAAAGCATGTAGGGAGAAGCTGCGGAGGGA
TAGGCTAAACGACAAGTTTTTGGAGCTGGGTTCTATTCTGGACCCTGGAAGGCCTCCTAAAACAGACAAGGCTGCTGTTTTGGTTGATG
CGGTTAAAATGGTGACTCAGTTACGGGGTGAAGCCCACAAGCTAAAGGACTCCAATTCAGATCTCCAACAGAAGATTAAGGAGTTGAA
GGCTGAGAAGAATGAGCTGCGTGATGAGAAGCATCGATTGAAGACTGAGAAGGAAAAGCTAGAGCAACAGATGAAAGGCATGAATG
CTCAACCCCAGTACCTCCCCCCACCTGCCATTCCCGCTGCATATACTGCTCAAGGTCAACCCGGCGGAAACAAGTTGGTGCCTTTCATT
GGTTACCCGGGAGTCGCTATGTGGCAGTTCATGCCACCCGCTGCGGTAGACACCTCTCAGGATCATGTACTCCGTCCCCCCGTTGCCTA
A

Table S4. Yeast one-hybrid experimental genes synthesis sequences in *Santalum album*

Transformation lists name	Lists（genes 5′→3′）
pAbAi-SSy1G-box-F	AATATAGAGTAACGTGCTAAGACTATAATATAGAGTAACGT GCTAAGACTATAATATAGAGTAACGTGCTAAGACTAT
pAbAi-SSy1G-box-R	ATAGTCTTAGCACGTTACTCTATATTATAGTCTTAGCACGTT ACTCTATATTATAGTCTTAGCACGTTACTCTATATT
pAbAi-mSSy1G-box-F	AATATAGAGTCCAACGCTAAGACTATAATATAGAGTCCAAC GCTAAGACTATAATATAGAGTCCAACGCTAAGACTAT
pAbAi-mSSy1G-box-R	ATAGTCTTAGCGTTGGACTCTATATTATAGTCTTAGCGTTGGACTCTATATTATAGT CTTAGCGTTGG ACTCTATATT
pAbAi-CYP1G-box-F	TTGATAGATATACGTGCACCTATAAGTTGATAGATATACGTGCACCTATAA GTTGATAGATATACGTGCACCTATAAG
pAbAi-CYP1G-box-R	CTTATAGGTGCACGTATATCTATCAACTTATAGGTGCACGTATATCTATCAACTTAT AGGTGCACGTATATCTATCAA
pAbAi-CYP2G-box-F	GTGTCCGAGCCACGTTACGGTTTGGGGTGTCCGAGCCACGTTACGGTTTGGGGTGT CCGAGCCACGTTACGGTTTGGG
pAbAi-CYP2G-box-R	CCCAAACCGTAACGTGGCTCGGACACCCCAAACCGTAACGTGGCTCGGACACCCC AAACCGTAACGTGGCTCGGACAC
pAbAi-CYP3G-box-F	TAATTTGAAGCACGTGAATTTGGCATTAATTTGAAGCACGTGAATTTGGCATTAATT TGAAGCACGTGAATTTGGCAT
pAbAi-CYP3G-box-R	ATGCCAAATTCACGTGCTTCAAATTAATGCCAAATTCACGTGCTTCAAATTAATGC CAAATTCACGTGCTTCAAATTA
pAbAi-CYP4G-box-F	TCCCACCCACGTAGCTTCGCCCACGTTTGGAATCATCCCACCCACGTAGCTTCGCC CACGTTTGGAATCATCCCACCCACGTAGCTTCGCCCACGTTTGGAATCA
pAbAi-CYP4G-box-R	TGATTCCAAACGTGGGCGAAGCTACGTGGGTGGGATGATTCCAAACGTGGGCGAA GCTACGTGGGTGGGATGATTCCAAACGTGGGCGAAGCTACGTGGGTGGGA
pAbAi-mCYP3G-box-F	AATTTGAAGTCAACGAATTTGGCATTAATTTGAAGTCAACG AATTTGGCATTAATTTGAAGTCAACGAATTTGGCAT
pAbAi-mCYP3G-box-R	ATGCCAAATTCGTTGACTTCAAATTAATGCCAAATTCGTTGACTTCAAATTAATGCC AAATTCGTTGACTTCAAATTA
pAbAi-mCYP4G-box-F	TCCCACCATTTAAGCTTCGCCTTAATCTGGAATCATCCCACCATTTAAGCTTCGCCT TAATCTGGAATCATCCCACCATTTAAGCTTCGCCTTAATCTGGAATCA
pAbAi-mCYP4G-box-R	TGATTCCAGATTAAGGCGAAGCTTAAATGGTGGGATGATTCCAGATTAAGGCGAA GCTTAAATGGTGGGATGATTCCAGATTAAGGCGAAGCTTAAATGGTGGGA

Supplementary Figures

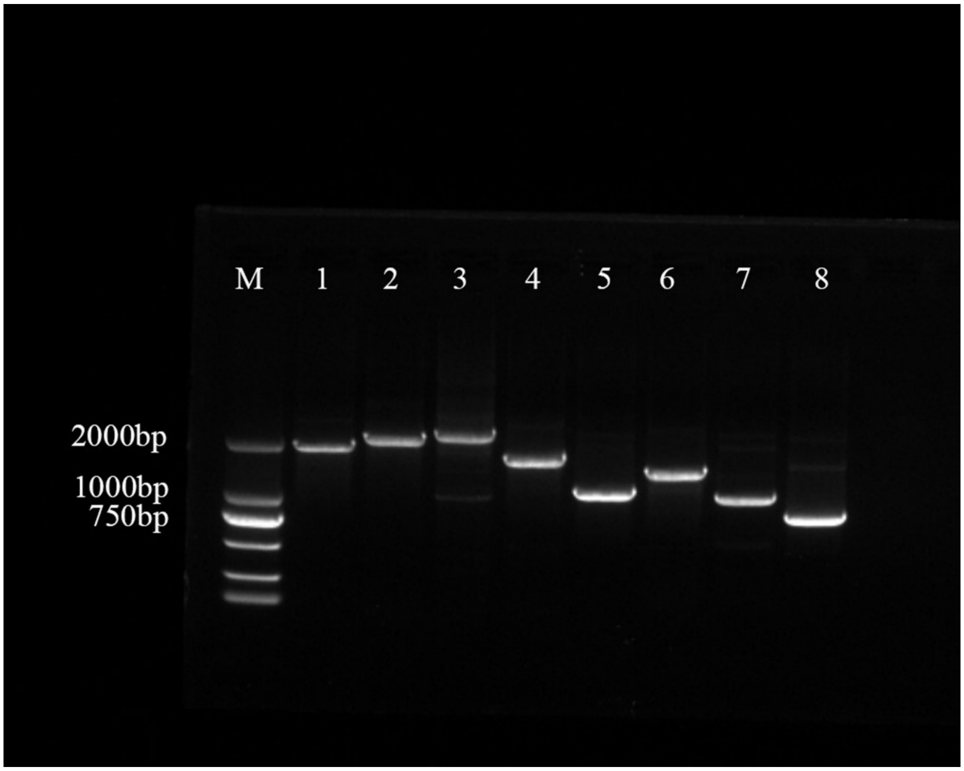

Figure S1. TF open reading frames (ORFs) were amplified by RT-PCR and then electrophoresed on a 0.1% agarose gel

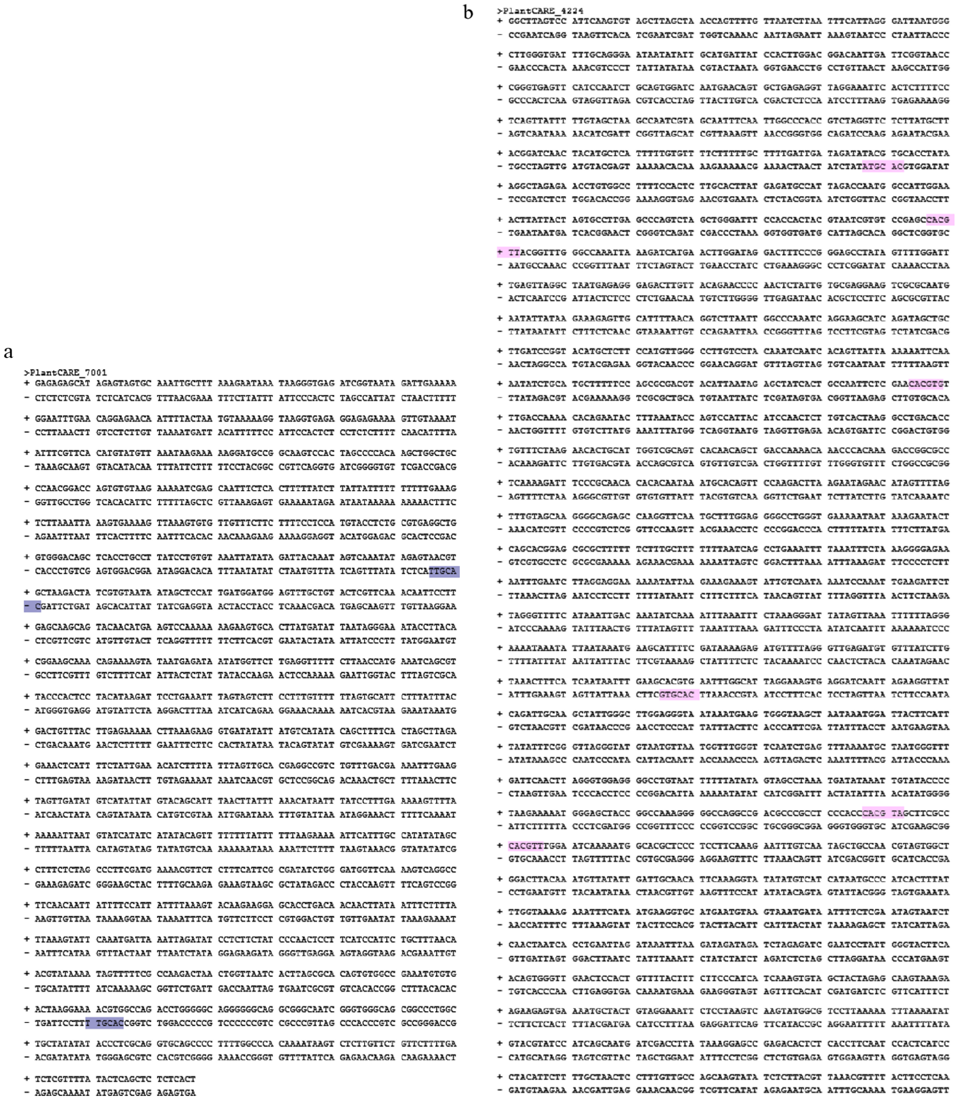

Figure S2. Detection of G-box elements of *SaSSy* ((**a**) G-box marked blue) and *SaCYP736A167* ((**b**) G-box marked pink) promoter.

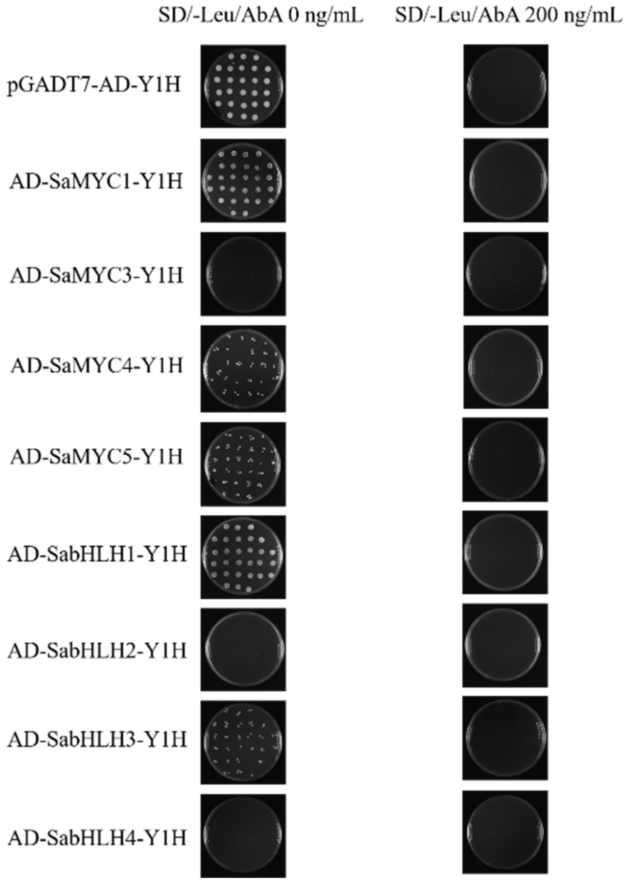

Figure S3. Transcription factor activity verification.

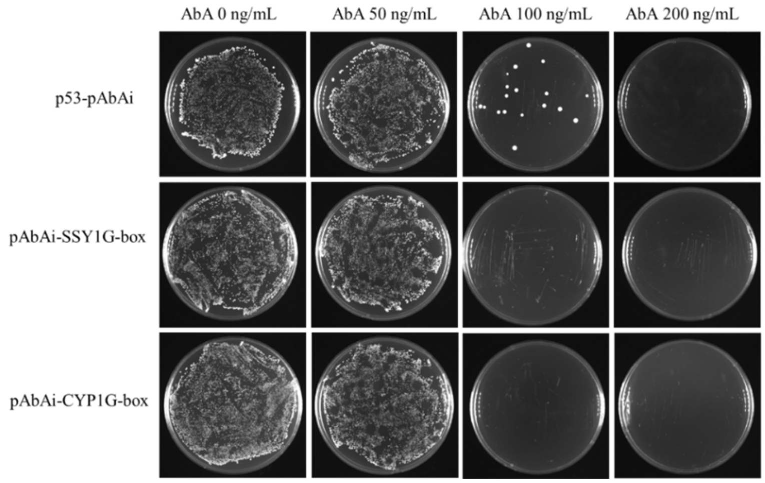

Figure S4. Screening of the inhibitory AbA concentrations of bait strain. Note: p53-AbAi was the positive control; pAbAi-SSy1G-box was the G-box element strain of *SaSSy*; pAbAi-CYP1G-box was the G-box element strain of *SaCYP736A67*; the SD/-Leu selection media with AbA concentration of 0 ng/mL, 50 ng/mL, 100 ng/mL, 200 ng/mL, respectively.

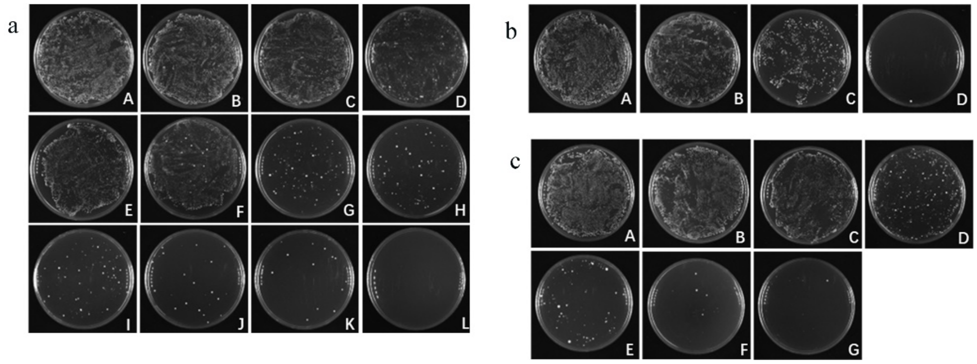

Figure S5. Screening AbA concentration of mutant G-box element bait strain. (**a**): Screening of the lowest inhibitory AbA concentration of mutant bait strain pAbAi-mSSy1G-box; (**b**): Screening of the lowest inhibitory AbA concentration of mutant bait strain CYP3G-box; (**c**): Screening of the lowest inhibitory AbA concentration of mutant bait strain pAbAi-mCYP3G-box. A-L: The SD/-Leu selection media with AbA concentration of 0 ng/mL, 50 ng/mL, 100 ng/mL, 200 ng/mL, 300 ng/mL, 400 ng/mL, 500 ng/mL, 600 ng/mL, 700 ng/mL, 800 ng/mL, 900 ng/mL, 1000 ng/mL, respectively.
